# The Goldilocks Approach: A Review of Employing Design of Experiments in Prokaryotic Recombinant Protein Production

**DOI:** 10.3390/bioengineering5040089

**Published:** 2018-10-19

**Authors:** Albert Uhoraningoga, Gemma K. Kinsella, Gary T. Henehan, Barry J. Ryan

**Affiliations:** Dublin Institute of Technology, Dublin D01 HV58, Ireland; C12712219@mydit.ie (A.U.); Gemma.Kinsella@dit.ie (G.K.K.); Gary.Henehan@dit.ie (G.T.H.)

**Keywords:** recombinant protein production, design of experiments, screening design, response surface methodology, process optimization

## Abstract

The production of high yields of soluble recombinant protein is one of the main objectives of protein biotechnology. Several factors, such as expression system, vector, host, media composition and induction conditions can influence recombinant protein yield. Identifying the most important factors for optimum protein expression may involve significant investment of time and considerable cost. To address this problem, statistical models such as Design of Experiments (DoE) have been used to optimise recombinant protein production. This review examines the application of DoE in the production of recombinant proteins in prokaryotic expression systems with specific emphasis on media composition and culture conditions. The review examines the most commonly used DoE screening and optimisation designs. It provides examples of DoE applied to optimisation of media and culture conditions.

## 1. Introduction

Advances in biotechnology, including the development of genetic engineering and cloning, have provided a means for the large scale expression of heterologous proteins for different applications [1]. Currently, recombinant proteins are widely used in the biological and biomedical industries as well as in research with their market share increasing rapidly [2,3]. The production of high yields of soluble and functional recombinant protein is the ultimate goal in protein biotechnology [4]. To achieve this objective, many key aspects such as the expression system, the expression vector, the host strain, the purification tag, the media composition, the induction conditions and the purification methods need to be carefully evaluated and optimised before embarking on large scale production of a recombinant protein of interest [5,6,7].

Although both eukaryotic and prokaryotic expression systems are used for overproduction of soluble recombinant protein, choosing the right system for your protein depends, amongst other things, on the growth rate and culturing conditions of host cells, the level of the target gene expression and post translational processing of the synthesized protein [8,9]. The most commonly used prokaryotic systems are based on expression in bacteria, including *E. coli* and *Bacillus* species [10,11]. There is no single method which is universally successful for protein expression that will ensure the production of a desired concentration of soluble and functional protein [12,13,14]. Varying factors that influence protein expression in a trial-and-error process to achieve optimum protein expression has been troublesome [15]. To overcome this problem, statistical approaches have been used to evaluate the variables that have the largest influence on the production of a recombinant protein of interest in terms of yield [16,17], product quality [18], purity [19,20] and solubility [21,22]. These statistical processes include the Design of Experiment (DoE) approach [23,24]. This approach advances the traditional one-factor-at-a-time (OFAT) method, which involves varying one factor while other factors are held constant. This single variable OFAT approach results in the need to run multiple experiments with a high risk of failing to identify the true optimum [25]. The DoE method provides for a significantly reduced experimental matrix [26,27,28].

There are an increasing number of published studies on the application of statistically based optimization processes in the field of protein biotechnology [18,29]. This has been matched by a corresponding increase in the application of DoE methods, such as screening and optimisation designs, to enhance protein production. This review examines the literature on the DoE methodologies commonly employed to evaluate the effect of media composition and culture conditions on recombinant protein expression. It will focus on the application of DoE to increase recombinant protein expression in prokaryotic systems, where high yields can be achieved but poor product quality remains a risk [30]. It also provides an overview of the important statistical analysis tools embedded in common DoE software. These tools facilitate the interpretation of experimental data which ultimately allows the identification of optimal factor levels for maximum yield. Finally, the review provides some thoughts on the benefits of the common DoE methods typically used in recombinant protein production in order to direct future research efforts.

## 2. Production of Recombinant Proteins in a Prokaryotic Expression System

### 2.1. Factors that Inform the Choice of Expression System

Protein purification from natural sources can require a large quantity of the source organism and may yield only small amount of target protein after several rounds of extraction and purification [4,31]. Recombinant expression of proteins has become an indispensable tool to produce proteins to satisfactory yields [32] and to meet the demands of industry and research [1,33]. With the aid of genetic engineering, a desired gene cloned into a suitable expression vector can be overexpressed as a recombinant protein of interest [34]. Recombinant proteins can be expressed in cell cultures of bacteria [35], yeasts [36], mammalian cells [37,38], plants [39] and insects [40]. However, the prokaryotic systems remains the most attractive hosts due to their low cost, high productivity and rapid production rates [30]. Prokaryotic heterologous protein expression is mainly carried out in the bacteria *E. coli*, although increasingly the *Bacillus* species are being employed [41,42,43]. Drawbacks of prokaryotic expression systems include poor protein quality, due to the inability of prokaryotic cells to carry out post-translational modifications such as glycosylation, the presence of toxic cell wall pyrogens, along with the formation of inclusion bodies resulting in aggregated and insoluble heterologous protein [44]. Some widely used bacterial expression systems that are commercially available are listed in Table 1.

While there are a variety of expression vectors commercially available, their choice is strongly based on the combination of replicons, promoters, selection markers, multiple cloning sites and fusion proteins [11]. An informed decision on the best expression plasmid [10,51,52,53,54] can be confusing. The most commonly used expression plasmids [22,55,56,57,58] and their key features such as promoters [59,60,61,62,63], affinity tags [64,65] and selection markers [7] have been extensively reviewed in the literature, primarily focusing on the *E. coli* prokaryotic expression system. Widely used *Bacillus* strains [66,67], vectors and promoters have also been reviewed [68,69,70].

### 2.2. Factors that Influence Media Composition and Culture Conditions in an Expression System

A careful selection of expression system, expression vector and host does not always guarantee the production of a large amount of target protein in soluble and active form [7]. Media composition and induction conditions have a significant influence on recombinant protein expression levels [71,72,73] and solubility [45]. For example, media containing a defined concentration of salts, peptone and yeast influences the yield of a recombinant glucosidase [47]; while media composition does not always have a major effect on protein solubility [51]. Prosthetic groups in media are known to prevent the formation of inclusion bodies [74] where required by the protein [41,75]. The most common media used in prokaryotic expression systems, along with their advantages and disadvantages, have been reviewed elsewhere [76]. Culture conditions are another set of factors that must be carefully optimised to achieve high yields of heterologous protein [14]. Factors such as cell density prior to induction, inducer concentration, induction temperature and induction duration are all known to influence yield [77,78,79,80,81].

### 2.3. Enhancing the Production of Recombinant Proteins in a Prokaryotic Expression System by DoE

**Figure 1:**
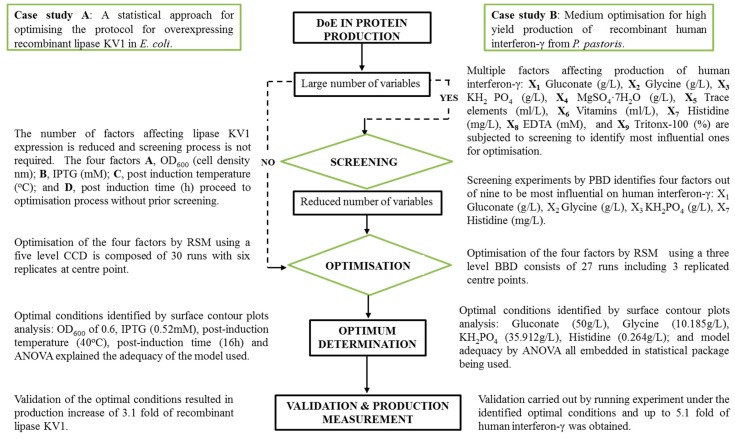
A typical DoE workflow in protein production. **Case study A** illustrates the optimization of recombinant lipase KV1 expression in *E. coli* [84] where a screening process was not required since the number of factors affecting this enzyme is not large (four factors). The four factors (A, B, C, D), therefore, underwent optimisation by Central Composite Design (CCD) under Response Surface Methodology (RSM) which resulted in a yield increase in protein expression of 3.1-fold. **Case study B** describes the optimisation process for high yield production of recombinant human interferon-γ [85]. In this case, the number of factors involved is large (nine factors) and they were subjected to a screening process before optimisation. Four factors (X_1_, X_2_, X_3_, X_7_) out of nine were identified by Plackett-Burman Design (PBD) based screening to be the most influential and subsequently used for further optimisation. A Box-Benkhn Design (BBD) also under RSM was selected to optimize the screened factors and increased the production of human interferon-γ up to 5.1 fold. Further details of these two case studies can be found in the references provided and similar cases are found in Tables 4 and 7.

It can be difficult to make informed decisions regarding the optimal combination of expression system, conditions and media components. Oftentimes this results in an unsatisfactory and costly trial-and-error process being employed to enhance the overall production yield [64]. To address this problem more effective, statistically supported, approaches have been developed and have gained significant traction. In this approach, a controlled model is developed defining media components, induction and expression conditions based on the recombinant protein of interest [16]. DoE, employed in this way, has provided powerful tools to screen and optimise factors affecting recombinant protein expression [82]. This is due to DoEs’ ability to identify factors affecting recombinant protein production and optimise the process with the minimum number of experiments [83]. A typical DoE workflow is depicted in diagrammatic form (see Figure 1). The desired output, or response, is to achieve a high yield of a protein of interest and involves three main stages:

Stage 1. The first stage of the process is to compile a list of factors that can influence protein expression. These are usually such factors as; induction temperature, induction duration, pH, media components (carbon source, nitrogen source, micronutrients).
Stage 2. At this stage, a suitable software package such as MINITAB, JMP or Design Experts will be acquired for the statistical analysis. The second stage of DoE aims to reduce the number of factors to a smaller subset, these being the most important factors (i.e. those with the greatest impact on expression). This process is known as screening. Having a smaller set of significant factors greatly simplifies the statistical process. Sometimes, if the number of factors is small (between 2 and 4) there is no need to carry out the screening stage. When looking at a factor that influences protein expression the concept of levels is important: temperature, for example, may be examined between 20 °C and 40 °C. These two temperatures represent the lowest and highest “level” of this parameter that will influence expression. For the purposes of modelling these two levels are input into the model for this factor. Similarly, the upper and lower levels are input for all other relevant parameters. It is important to note that the levels are input into the DoE package as +1 (highest value of a parameter) and −1 (lowest value of a parameter). This “coding” is carried out to avoid the use of multiple different measurement units for parameters such as pH, temperature. The software will then suggest a minimal set of experiments to explore the significance of each factor. The design of the experimental matrix can be selected from a range of choices such as Full Factorial Design, Plackett Burman Design or indeed a custom design. The objective is to assess the “main effect” of a factor (its direct effect on a response) as well as its “interaction effects” (the effect on other factors). The suggested experiments are carried out and the results are used to inform the next stage of the process—optimisation.
Stage 3. The final stage of the process is optimisation and is typically carried out with a set of three to four factors. An experimental RSM (Response Surface Methodology) design strategy is selected and experiments are run as for the screening stage. The optimisation process expresses the response surface as a polynomial and uses the input data to estimate its coefficients. The derivative of this polynomial is used to obtain inflection points corresponding to maxima or minima in the model. The model can be evaluated by looking at the goodness of fit between the model and experimental data. Finally, experiments using the optimum conditions predicted by the model are carried out to validate the model.

## 3. Design of Experiments (DoE) to Optimise Recombinant Protein Production

### 3.1. DoE; a Brief Overview

DoE is a statistical technique used to plan experiments and analyse data using a controlled set of tests designed to model and explore the relationship between factors and observed responses [14]. This technique allows the researcher to use the minimum number of experiments, in which the experimental parameters can be varied simultaneously, to make evidence based decisions [86]. It uses a mathematical model to analyse the process data, such as protein expression levels [87]. The model allows a researcher to understand the influence of the experimental parameters (inputs) on the response (outputs) and to identify a process optimum [88]. Furthermore, DoE software uses three-dimensional surface and contour plots, to visualise and understand the relationship between factors and responses [55,89]. In recombinant protein production, a DoE approach can significantly improve the efficiency in screening for most influential experimental parameters (e.g., media composition, culture condition etc.) and determine optimal experimental conditions [90].

The mathematical models employed in DoE define the process under study [91]. Screening designs such as Plackett Burman Design are based on a first order model [92] as shown in Equation (1).

(1) Y=β0+ΣβiXi

where Y is the response, β0 is the model intercept, βi is the linear coefficient and Xi is the level of the independent variables. A statistically significant level of 5% (*p*-value = 0.05) is commonly used to identify the most influential factors. The significance level (or *p*-value) of each variable is based on its effect on the response and is calculated using Student’s *T*-test [85] in Equation (1).

(2) $$t_{xi}=\frac{E(X_{i})}{S.E.}$$

where E(*X_i_*) is the effect of variable *X_i_* and S.E., the associated standard error. Factors with *p*-value < 0.05 are statistically significant while factors with *p*-value > 0.05 are not statistically significant (see Table 5 for more details). Statistically significant factors are subjected to further optimisation by Response Surface Methodology. A second-order polynomial equation in which independent variables are coded using Equation (3) is used to input factors into the model (see Section 5.4.1).

(3) $$x_{i}=\frac{\left(Xi-X_{cp}\right)}{∆Xi},\;i=1,\;2,\;3\ldots k$$

where *x_i_* is a dimensionless value of an independent variable; *Xi* is real value of an independent variable; *X_cp_* is real value of an independent variable at the design centre point; and ∆*Xi* is step change in the real value of the variable *i* [93]. Replicates at the central point are required to check for the absence of bias between sets of experiments. The fit of the model is then evaluated through analysis of variance (ANOVA) which determines the significance of each term in the equation and estimates the goodness of fit in each case [94] (see Figure 5 and Table 9 for more details).

### 3.2. DoE Versus One-Factor-At-a-Time (OFAT)

**Figure 2:**
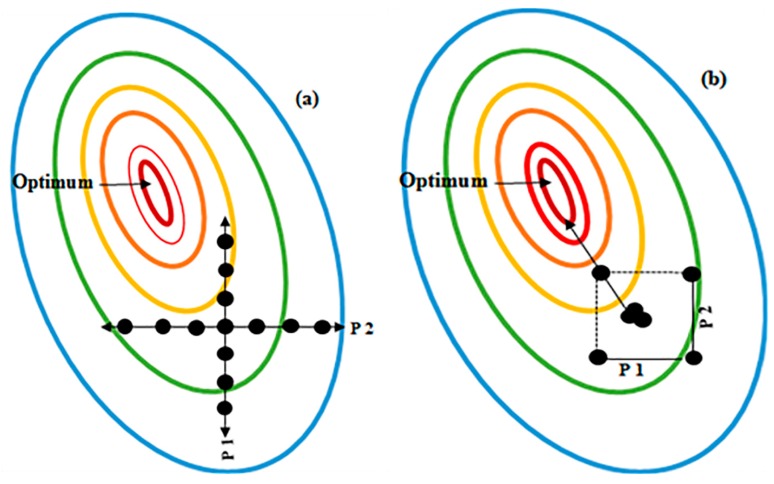
Comparison between Design of Experiments (DoE) and One-Factor-at-A-Time (OFAT) by examining the effect of two parameters, P1 (Parameter 1) and P2 (Parameter 2). (**a**) OFAT is performed using more experiments than DoE (each black dot represents an experiment) and does not identify the true optimum (indicated as a red oval). However, with the DoE approach (**b**) fewer experiments are used and the likelihood of finding the optimum conditions (in red) for the process being studied is high. With DoE the combined or interaction effect of P1 and P2 on the response can be identified and measured. The ovals indicate production yields, blue indicates the lowest yields, whereas red indicates highest yields, where the optimum is found. The DoE approach also identifies a pathway to the optimum response (indicated by the arrow).

DoE advances the traditional OFAT approach; OFAT fails to account for variables interacting with and influencing, each other and also requires significantly more experiments to converge on an optimum; all of which increases cost and time [95]. Figure 2 provides a brief comparative description between DoE and OFAT.

In recombinant protein expression, where various independent variables do not always act in isolation, it is likely that their interaction effects can significantly influence protein production [96]. Therefore, it is necessary to use a controlled set of tests that can examine the effects of many interacting factors to achieve optimal expression [97].

## 4. Defining a DoE Workflow to Optimise Recombinant Protein Production

**Figure 3:**
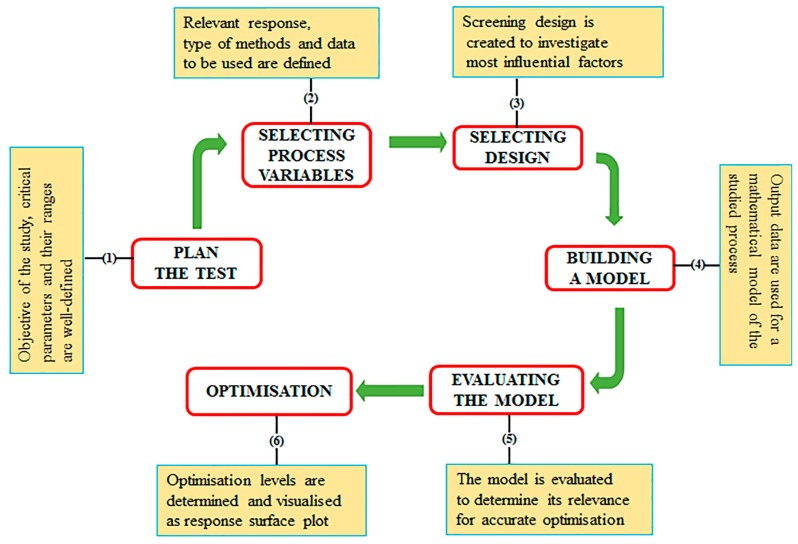
A typical DoE workflow for the optimisation of recombinant protein production. The figure describes the main steps involved in the experimental design when both screening and optimisation designs are used. (1) The objectives of the study are defined including the selection of factors, levels and responses. (2) Process variables and expected responses are identified; the process variable levels (for a 2 level study) are set as high (+1), low (−1), (on occasion a 0 point is included). (3) The experimental screening design is selected based on the objectives of the study and the number of factors involved. (4) A mathematical model is built with certain conditions to meet the desired objectives (e.g., measurement of all the desired responses, process stability and accurate approximation by polynomial models). (5) The response data are analysed and visualised using plots for ease of data interpretation. At this stage, a reduced number of factors (i.e., the most influential) are retained for the subsequent optimisation phase. (6) Further optimisation can be carried out (via an optimisation DoE design).

Employing DoE to optimise the production of a recombinant protein can be divided into two main work packages, initial screening and subsequent optimisation. To evaluate all the factors that influence a production process, it is initially required to carry out a wide-ranging experimental screening. This first screening step will identify all factors that significantly influence recombinant protein production [98]. The second step in the workflow is to use a DoE optimisation design to achieve optimum production focusing only on the factors identified through the initial screening design. A variety of DoE software packages such as MINITAB (Minitab Ltd., State College, PA, USA), JMP (SAS Institute, Cary, NC, USA) and Design Experts (Science Plus Group, Groningen, the Netherlands) are commercially available and provide a variety of factorial designs depending upon the objective of the experiment. Regardless of the statistical package used, the main steps of a typical DoE workflow include planning the test, screening and optimisation (detailed schematically in Figure 3).

## 5. A Suggested DoE Workflow for Recombinant Protein Production

### 5.1. Planning the Test; Selection of Factors and Associated Levels Influencing Recombinant Protein Production 

The DoE workflow in protein production, like in any other DoE process optimisation, starts with the planning the test [99]. This involves defining the objective of the study, identifying factors involved and associated levels (i.e., high, central and low). Thus, preliminary experiments are recommended when knowledge of effects of factors on the experiment is not sufficient to set levels. The factors are input parameters that can be modified in the experiment and are referred to as the controllable factors. The levels of factors are fixed based on their working limits [82]. The most popular experimental designs are two level designs although more levels can be used depending upon the type of design and objective of the study. Table 2 depicts a two level experimental design.

In general, for recombinant protein expression subjected to DoE, the most commonly selected factors relate to media composition and include components such as yeast extract [100], K_2_HPO_4,_ MgSO_4_, starch, glucose, peptone, NaCl, sucrose, glycerine [101]. For induction conditions, common factors selected are incubation time, incubation temperature, pH, agitation, inoculum age and size [102,103]; induction period, induction temperature, culture inoculation concentration [48,104]; Optical Density (OD), Isopropyl β-D-1-thiogalactopyranoside (IPTG) concentration [21].

### 5.2. Screening Designs to Identify Factors that Significantly Affect Recombinant Protein Expression

Screening designs are used to devise a matrix using factors and levels as formulated in the planning stage. [105]. By employing the statistical tools embedded in the DoE software, screening designs establish the relationships between variables and responses. The interaction effects between variables on a given response are also investigated [106]. In protein biotechnology, screening designs are mainly utilised to identify media composition and culture condition factors that significantly influence protein production [107]. Various researchers have explored the effects of both media components [94,107,108,109,110] and culture conditions [111,112] on protein expression. There are many different types of screening designs and their choice depends upon the nature of experiment and the objective of the study. The classical screening designs include Full Factorial Designs, Fractional Factorial Designs and Plackett-Burman Designs. Current DoE software, such as JMP from the SAS Institute, provides additional screening designs such as Definitive Screening Designs and Custom Designs. The most common screening designs are compared in Table 3.

#### 5.2.1. Full Factorial Design 

When little is known about the effects of the factors on a response, a full factorial design is recommended. This design includes all combinations of all factor levels and provides a predictive model that includes the main effects and all possible interactions [113]. This design consists of two, or more, levels with experimental runs that encompass all possible combinations of these levels, across all factors. In a full factorial design where k represents number of factors; 2^k^ represents the number of experiments required to carry out a two level design with k factors. Similar to other screening designs, Full Factorial Design can include centre points, randomisation and blocking variables to improve the efficiency of the design [14]. This approach was significant in screening for the most influential factors affecting recombinant protein production for a variety of proteins [114,115] (see Table 4).

#### 5.2.2. Fractional Factorial Design (FFD)

FFD is a recommended screening design when a large number of factors are involved. This design consists of reducing the initially large number of potential factors to a subset of the most effective ones and is represented using the following notation:

$$2\begin{array}{c} k-p\\ R \end{array}$$

where 2 represents number of levels, k the number of factors, p the extra columns required and R the resolution of the method. The method resolution describes the degree to which the estimated main effects are aligned with the estimated interactions associated with levels [22,116,117].

#### 5.2.3. Plackett-Burman Designs (PBD)

PBD design is often used as an alternative to fractional and full factorial designs because of its potential to reduce the gaps found in fractional designs and to strengthen the estimation of the main effects, which may have been disregarded when full factorial designs are used [118,119,120,121,122].

#### 5.2.4. Definitive Screening Design (DSD) and Custom Design (CD)

DSD and CD are a class of screening designs that have potential applications in recombinant protein expression for assessing the impact of a large number of factors on a given response. DSD has recently been reported to be particularly advantageous as it allows the estimation of the main effects of certain components alone but also the interactions between components as well as the factors with non-linear effects such as quadratic effects (an interaction term where a factor interacts with itself); all executed with the minimum number of experimental runs [123]. CD enables tailoring a design, whilst simultaneously minimising resource usage: it is highly flexible and more cost-effective than other screening designs. It allows for the best use of the experimental budget and tackles a wide range of challenges with the capability to model effects including centre points and replicates. However, in most cases this design allows for the estimation of main effects only. Table 4 summarises the most common screening designs, along with their roles in identifying most influential independent factors, in recombinant protein production.

The rationale of screening designs lies in identifying the variables that are statistically significant in influencing protein production among a large number of potentially important variables [128,129]. Table 5 illustrates how screening analysis identifies statistically significant factors based on their effect and probability values.

The screening process identifies most influential factors on the process under investigation (i.e., X_1_ and X_6_ in the example shown in Table 5) and thus paves the way for effective optimisation by reducing the number of factors to be optimised in the third work package of the DoE workflow [130].

### 5.3. Optimisation Designs to Maximise Recombinant Protein Production in Prokaryotic Systems

**Figure 4:**
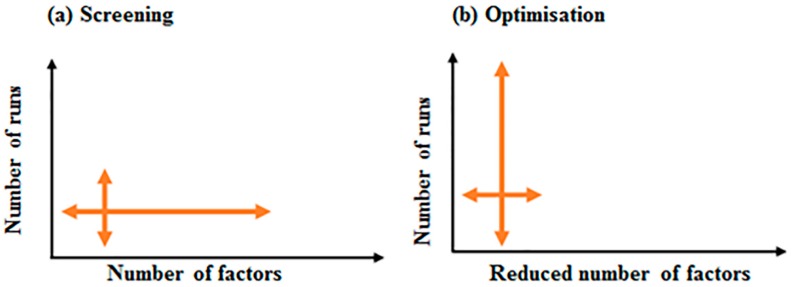
A comparative illustration of screening and optimisation designs. (**a**) In screening designs a large number of factors, with reduced number of runs, are used to screen for important factors affecting the process. (**b**) In optimisation designs, a reduced number of factors, with large number of runs, are utilised to find the optimum conditions for high yield of recombinant protein.

As a collection of statistical design and numerical optimisation techniques [131], optimisation uses the reduced number of variables identified in the previous screening process and focuses on finding the variable levels that result in an optimal yield [132,133]. Figure 4, describes the benefit of carrying out an optimisation process after a screening process has identified a small number of key variables.

Response Surface Methodology (RSM) is the most popular optimisation method [134]. It consists of mathematical and statistical techniques used to build empirical models capable of exploring the process space and studying the relationship between the response and process variables to find the optimal response [99,133,135]. In general, for a given number of factors, RSM requires more runs than screening designs, thus, the number of factors to consider should initially be reduced through an appropriate screening process. Central composite designs (CCD) and Box-Behnken designs (BBD) are the two of the major Response Surface Designs commonly used in recombinant protein optimization [136].

#### 5.3.1. Central Composite Design (CCD)

CCDs are favoured in process optimisation due to determine the coefficients of a second-degree polynomial which fit a full quadratic during response surface analysis [127]. CCD has been widely used in optimising protein production process specifically addressing the aim of increasing productivity and solubility [137]. There are different types of central composite designs such as uniform precision, orthogonal/block and so forth. However, a common standard characteristic includes the number of runs per design [138], which depends on the number factors (see Table 6). Central composite uniform precision designs are used to provide protection against bias in the regression coefficients while central composite orthogonal designs can be used to avoid correlations between coefficients of variables [139].

#### 5.3.2. Box Behnken Design (BBD)

BBDs are also a class of response surface designs; however, they differ from CCD in their design structure. For example, a CCD with 4 factors requires 31 runs (experiments), whereas a BBD only has 27 runs for the same number of factors. For 5 factors, CCD has 52 runs while BBD has 46 runs. Reduced runs can result in significant time and cost savings in an optimisation process. In optimisation experiments BBD is widely used as a good design to fit the quadratic model with fewer experiments [141]. Several studies show that BBDs have contributed to production increases for recombinant proteins (see Table 7).

#### 5.3.3. Summary and Choice of Optimisation Methods

Both CCD and BBD optimisation methods are widely used, the choice depends on the number of factors and objectives of the study (see Figure 1). The standard characteristic is that all response surface designs feature a second-order polynomial model to describe the process where interaction terms introduce curvature into the response function and a first-order equation is inadequate to fit the model [159,160]. CCD is the most preferred RSM [16,161] due to the fact that this design contains full factorial or fractional factorial modes, with the potential to add central points to evaluate the experimental error and axial points to check the variance of the model [14,140]. The number of runs (N) in CCD is calculated using Equation (4).

(4) $$N=k^{2}+2k+\mathrm{Cp}$$

where k is the number of factors and Cp the number of centre points [162]. Table 8 is an example of a two level CCD with two centre point replicates along with responses such as actual, predicted and residues (see Table 8).

### 5.4. Analysis and Interpretation of Optimisation Data

**Figure 5:**
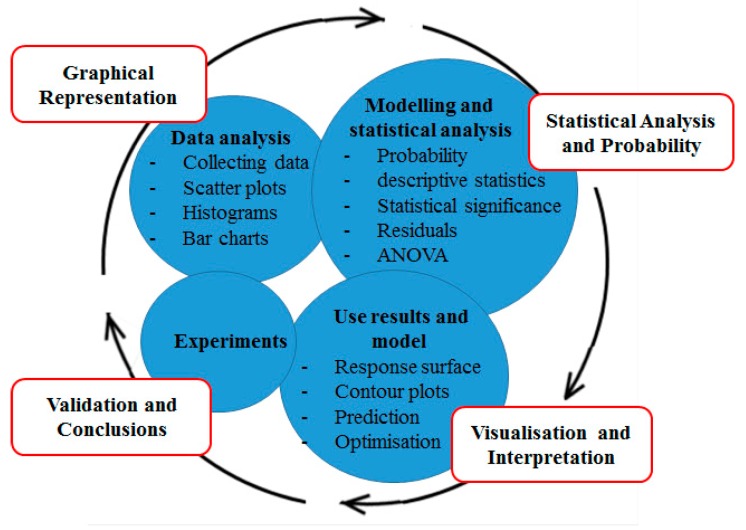
A typical DoE analysis route from initial Experiments to validation and conclusions. The rationale for data analysis is to evaluate the effects of variables on response. Graphical Representation shows how the data are distributed. The Statistical Analysis and Probability stage identifies variables that are statistically significant. This will identify variables that are important to bring forward to the subsequent optimisation step based on their statistical significance. The Visualization and Interpretation stage will focus on representational analysis that identifies optimal levels.

Regardless of the DoE design employed, the goal is to provide a methodology for conducting controlled experiments with the aim of identifying the vital process inputs and investigating interactions between them [163]. At a screening level, after the experimental data are entered, the DoE software generates a variety of graphs that are used to interpret the results obtained. These may be scatter plots, histograms, bar charts and Pareto charts that allow the researcher to identify the distribution of the data and statistical significance of the variables tested [85]. Different screening analysis methods have been used in the field of protein production [77,92,112,164]. Figure 5 illustrates a typical DoE data analysis and interpretation route from data visualisation, through experiment validation to conclusion.

#### Evaluation of Experimental Design and Predictive Model Validation 

For RSM analysis, the goals are to (i) develop a predictive model that describes how the process inputs influence the process output and (ii) determine the optimal settings of the inputs [165,166]. Following the completion of the optimisation experiments, the results are used to fit a second-order polynomial equation (Equation (5)) [85].

(5) $$\mathrm{Yi}=\beta_{0}+\sum \beta_{i}x_{i}+\sum \beta_{ii}{x_{i}}^{2}+\sum \beta_{ij}x_{i}x_{j}$$

where Yi is the predicted response, β_0_, β*_i_*, β*_ii_* and β*_ij_* are regression coefficients for the intercept, first-order model coefficients, quadratic coefficient and linear model coefficient for the interaction respectively [167,168]. The fit of the model is then evaluated through analysis of variance (ANOVA, Table 9) which compares the variation due to the change in the combination of variable levels with the variation due to the random errors [14,169].

The coefficient value of *R^2^* defines how well the model fits the data. The closer the *R^2^* is to 1, the better it describes the experimental data [21]. The Adjusted *R^2^* is used to check the adequacy of the model by measuring the amount of variation about the mean derived from the model; the closer the value is to 1, the better it describes the model [130]. For example, in Table 9, the R^2^ = 0.9971 indicates the significance of regression of the fitting equation and therefore, adequacy of discrimination, indicating that only 0.29% of the total variation could not be explained by the fitting equation [142]. When *R*^2^ = 99.71%, Adj-*R*^2^ = 99.63%, Pred-*R*^2^ = 99.48% are in good agreement with each other (as in Table 9), this provides confidence in the accuracy of the model [156].

**Figure 6:**
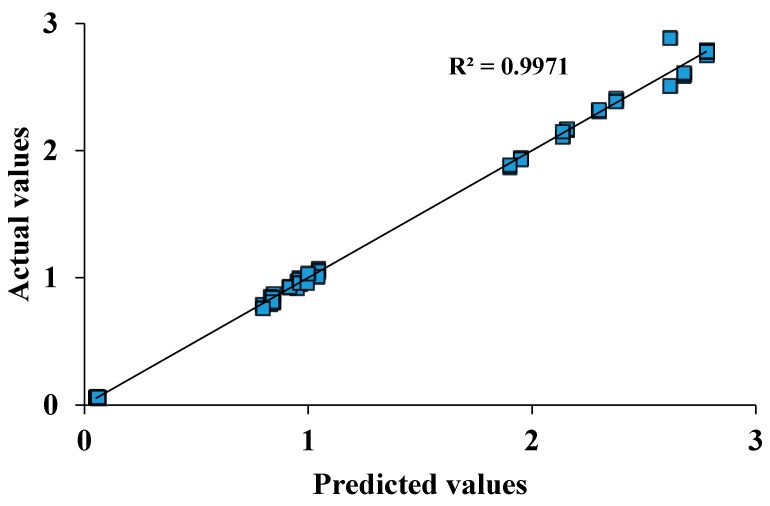
A linear plot estimating accuracy of a regression model by comparing actual versus predicted data sets. The plot determines the correlation between the model’s predictions and actual data and thereby indicates how well the model fits the data. The closer the value of *R^2^* is to 1, the better the fit of the line to the data and the goodness of the model.

Additionally, the *p*-value and signal-to-noise ratio are used to estimate the quality of the model. For a significant model, a *p*-value < 0.05 is desirable [170]. Appropriate precision measures the signal-to-noise ratio; where a ratio greater than 4 indicates an adequate model [171] and is commonly used in protein production optimisation [172,173]. Furthermore, the *p*-value lack of fit and the plot of observed values versus predicted values are used to estimate the quality of the model. With a good model, the *p*-value lack of fit should be >0.05 [168] as shown in Table 9. Finally, all data should fall on the straight line on the observed versus predicted plots [145] as shown in Figure 6.

### 5.5. Optimum Determination

**Figure 7:**
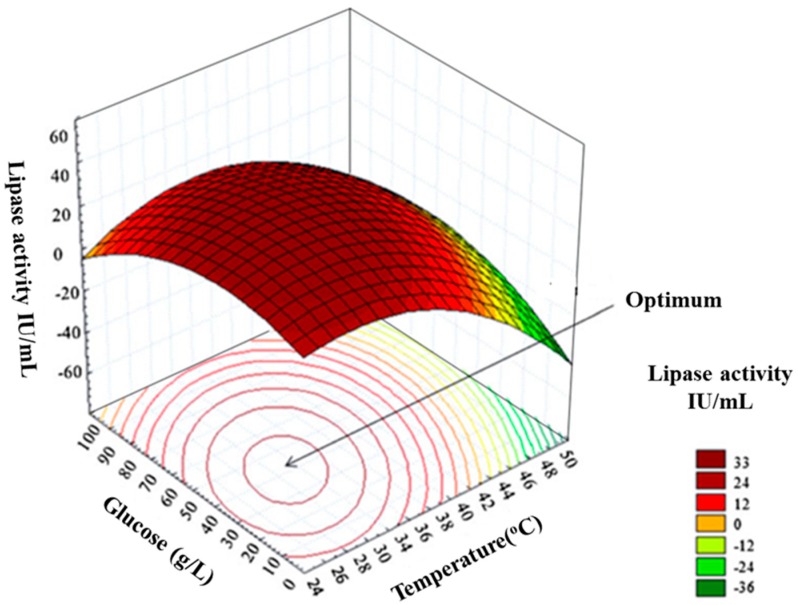
An example of response surface and contour plot adapted from Nelofer et al., 2012 [163]. The figure depicts the two-factor interaction (in this case the two factors explored are glucose and culturing temperature) where one factor influences the response of another factor. It also shows the visualisation of optimum levels. The colour scale indicates the level of lipase activity (IU/mL) where red indicates the region of optimal yield, yellow indicates medium yield, and green indicates low yield. In this case, the optimal enzyme activity (33 IU/mL) was achieved at a culture temperature between 30 °C and 34 °C; and a glucose concentration between 40 g/mL–50 g/mL. Image used with permission.

Once the predictive model has been validated, it can be used to determine the optimised parameters. The statistical tools embedded in DoE software are used to generate 3D-graphs, called surface contour plots that visually describe the relationship between variables and response [174,175]. The 3-D surface and contour graphs are generated as a combination of two test variables with the others maintained at their respective zero levels [176] see Figure 7. Surface, contour and residual plots, along with ANOVA, are the main optimisation analysis tools commonly used to determine optimum levels for high yields of recombinant protein [20,177,178,179].

## 6. Conclusions; Getting It ‘*Just Right*’

DoE offers many choices for screening and optimisation designs which advance traditional optimisation methodologies, such as one-factor-at-a-time. The statistical approach offered by DoE has proven to be applicable in protein biotechnology effectively investigating media composition and culture condition factors in recombinant protein production. DoE’s ability to identify the most influential factors in recombinant protein expression through screening designs and identify the factor/levels that give the maximum yield has considerably enhanced the production of soluble, active recombinant protein. With the recent development of more flexible screening and optimisation designs and enhancements in computational processing DoE will continue to find applications in biotechnology; in recombinant protein production and beyond.

## Figures and Tables

**Table 1:** Summary of the most widely used recombinant expression strains from *E. coli* and *Bacillus* species outlining their advantages and disadvantages.

	General Advantages	Disadvantages	References
Most common *E. coli* strains	Rapid expression, high yield, ease of culture and gene modification, cost effective.	Post translational modification not possible. Inclusion body formation	[41,45,46]
BL21, B21-Codonplus (RIL), BL21(DE3), BL21(DE3)pLys S/E, BL21 Star, C41(DE3), C43(DE3), Codon plus (RP), Lemon21(DE3), M15, Origami, Rosetta, SG13009, Shuffle Derivatives of K-12, AD494 and HMS174.			
Most common *Bacillus* species			
*Bacillus brevis, Bacillus megaterium and Bacillus subtilis.*	Preferred for homologous expression of some enzymes (e.g., proteases and amylases), Strong secretion, no involvement of intracellular inclusion bodies and ease of manipulation.	Contains proteases, which may hydrolyse recombinant proteins.	[42,47,48,49,50]

**Table 2:** An example of a two level experimental design having nine factors that are known to influence recombinant protein expression. In this case the nine factors relate to two experimental components; media composition and induction conditions. When planning the screening phase the selected factors (yeast extract, tryptone, glycerol, NaCl, Inoculum size, IPTG concentration, induction temperature, incubation time and pH, labelled X_1_ to X_9_ respectively) and associated levels (high, defined as +1 and low defined as −1 are selected to cover the intended experimental space (i.e., to cover the productive range). The levels are defined as the range between the known working limits.

	Factors	Levels	
		Low	High
Media composition	X_1_ Yeast Extract	−	+
	X_2_ Tryptone	−	+
	X_3_ Glycerol	−	+
	X_4_ NaCl	−	+
Induction condition	X_5_ Inoculum size	−	+
	X_6_ IPTG concentration	−	+
	X_7_ Induction temperature	−	+
	X_8_ Incubation time	−	+
	X_9_ pH	−	+

**Table 3:** A comparison of DoE screening designs commonly used in optimizing recombinant protein production. The table lists the types of screening designs; the effect explained by the model along with number of factors and associated number of runs (a rune refers to an experiment). It should be noted that extra runs (such as those related to central points) can be added when required. Custom design is more flexible and allows the designer to select the number of experimental runs.

		Factors					
		Number of Runs					
Screening Design	Effect explained by the model	2	3	4	5	6	7
Full Factorial Design	Main effect and 2 factor interactions	4	8	16	32	64	128
Fractional Factorial Design	Main effect only	-	-	-	8	8	8
	Main effect and 2 factor interactions	-	8	8	16	16	16
	Main effect and 2 factors interactions	-	-	16	16	32	64
Plackett-Burman Design	Main effect only	-	-	-	-	12	12
Definitive Screening Design	Main effect and 2 factor interaction	-	13	13	13	13	17
	Main effect, 2 factor interaction and quadratic effects	-	17	17	17	17	22
Custom Design	Main effect only	≥3	≥4	≥5	≥6	≥7	≥8

**Table 4:** A selection of the widely used screening designs and their application in identifying the influential factors on the production of recombinant proteins.

Host Organism	Protein Involved	Screening Design	Factors Studied	Screened Significant Factors	Reference
*Bacillus I-1018*	Xylanase	Full Factorial Design	Media composition	Xylan, casein hydrolysate, NH_4_Cl	[114]
*E. coli*	Non-structural protein NS3	Full Factorial Design	Culture condition	temperature, induction length	[124]
*Pseudoalteromonas IND11*	Fibrinolytic enzyme	Full Factorial Design	Media composition	pH, maltose and NaH_2_PO_4_	[115]
*E. coli*	Zinc-metalloprotease (SVP2)	Fractional Factorial Design	Media composition and culture condition	IPTG and Ca^2+^ ion concentration and temperature	[22]
*E. coli*	Soluble pneumolysin	Fractional Factorial Design	Media composition and culture condition	Temperature, tryptone and kanamycin	[6]
*Bacillus cerius*	L-asparaginase	Plackett-Burman	Media composition	Soya bean meal, asparagine, woodchips, NaCl	[122]
*E. coli*	Vascular endothelial growth factor	Plackett-Burman design	Media composition and culture condition	Glycerine, inducing time, peptone	[125]
*P. aeruginosa*	L-asparaginase	Plackett-Burman Design	Culture condition	pH, casein hydrolysate and corn steep liquor	[126]
*P. pastoris*	Human interferon gamma	Plackett-Burman Design	Media composition	Gluconate, glycine, KH_2_PO_2_	[85]
*S. griseorubens*	Chitinase	Plackett–Burman Design	Media composition	Yeast extract and K_2_HPO_4_, KH_2_PO_4_	[127]

**Table 5:** Identification of the statistically significant factors during a screening process using a Fractional Factorial Design. The table depicts the effect, positive or negative and *p*-value for seven factors examined (labelled X_1_ to X_7_ respectively). The effect of each factor, positive (+) or negative (−) is identified during the analysis stage using the statistical formula imbedded in DoE software used (JMP in this example). Interaction effects are also identified (e.g., X_5_*X_1_ and X_3_*X_7_; where * indicates an interaction). The *p*-value of each factor is also shown, at the significance level of 0.05. In this example, the highlighted factors, (X_3_, X_6_, X_1_), were identified as the most influential based on their high effects (−1.11273, 0.2252, 0.17492) and *p*-values < 0.05 (0.001, 0.0143, 0.0296). Thus, only factors X_3_, X_6_ and X_1_ are statistically significant at the level of 0.05, with X_3_ having a negative effect while X_6_ and X_1_ have positive effects. Other factors, X_2_, X_4_, X_5_, X_7_ and interactions X_5_*X_1_, X_3_*X_7_ are not statistically significant.

Factor	Effect	Relative Effect	*p*-Value
X_3_	−1.11273	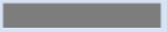	0.001
X_6_	0.2252	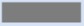	0.0143
X_1_	0.17492	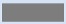	0.0296
X_4_	0.06408	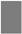	0.2215
X_7_	0.04154	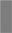	0.4112
X_2_	−0.07970	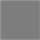	0.1421
X_5_ X_5_*X_1_ X_3_*X_7_	0.00233 0.04153 −0.06405	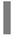 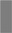 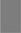	0.9664 0.4211 0.2623

**Table 6:** Common CCD components and the possible total number of runs. Factorial, axial and central points are the main components of a typical CCD and the total number of runs is dictated by the number of factors being tested. As the number of factors increases, the number of component points increase and so the total number of runs. In some cases, CCDs do not contain axial points, especially when the variance of model prediction is not suspected [140].

Number of Factors	Number of Factorial Points	Number of Axial Points	Number of Central Points	Total Number of Runs
2	4	4	5	13
3	8	6	6	20
4	16	8	7	31
5	16	10	6	32
6	32	12	9	53
7	64	14	14	92

CCD has been extensively used to optimise the production of recombinant proteins (see Table 7).

**Table 7:** RSM methods used to optimise the production of recombinant proteins along with their effect on yield and citing reference.

Microorganism	Recombinant Protein	RSM Methods	Optimised Factors	Optimised vs. Non-Optimised Yield	Reference
*E. coli* BL21	Superoxide dismutase	Box–Behnken design	Tryptone, tween-80, lactose	Enzyme activity increase by 1.54-fold	[142]
*E. coli* BL21-SI	Human interferon beta	Box–Behnken Design	Temperature, cell density, NaCl	hIFN- β concentration increase by 5-fold	[143]
*E. coli* BL21-SI	Human interferon gamma	Box–Behnken Design	Temperature, biomass concentration, NaCl	hIFN- γ concentration increase by 13-fold	[144]
*P. pastoris GS115*	β-glucosidase	Box-Behnken Design	Sorbitol, MeOH, pH	Enzyme activity increase by 3.3-fold	[145]
*Bacillus circulans* GRS 313	Amylase	Central Composite Design	Soybean meal, yeast extract, wheat bran	Enzyme yield increase by 1.25-fold	[146]
*Bacillus* IMG22.	*α*–amylase	Central Composite Design	Starch, yeast extract, glycerol, peptone	Enzyme activity reached 17.54 IU/mL	[147]
*E. coli BL21(DE3), Rosetta 2 (DE3), Rosetta blue (DE3), and Rosettagami2(DE3)*	Cyclodextrin glucanotransferase	Central composite Design	IPTG, arabinose B, post induction temperature	Enzyme activity increase by 3.45-fold	[148]
*E. coli DH5α*	Cytochrome 2C9 protein	Central Composite Design	Ampicillin, chloramphenicol, IPTG, peptone	Enzyme production increased by 1.05- fold	[149]
*E. coli* BL21 (DE3)	Interferon beta	Central Composite Design	DCW (dry cell weight), IPTG	Production increase more than 3-fold	[137]
*E. coli* BL21 (DE3)	L-Asparaginase	Central Composite Design	Tryptone, yeast extract, peptone, CaCl_2_	Enzyme activity reached 17,386 U/L	[150]
*E. coli* BL21	Peptide T-20	Central Composite Design	NPK, IPTG, post induction time	Production increase by more than 2-fold	[106]
*E. coli* BL21 (DE3)	TaqI endonuclease	Central Composite Design	Glucose, (NH_4_)_2_HPO_4_, KH_2_PO_4_, MgSO_4_.7H_2_O	Enzyme yield increase by about 3.6-fold	[151]
*E. coli DH5α*	Xylanase	Central Composite Design	Glucose, (NH_4_)_2_HPO_4_, CK_2_HPO_4_, DKH_2_PO_4_, MgSO_4_	Production increase by 1.7- fold	[152]
*E. coli* BL21	Bromelain	Central Composite Design	Temperature, inducer concentration, post induction period	Enzyme activity increase by 1.3-fold	[153]
*E. coli* BL21	Phytase	Central Composite Design	Tryptone, yeast extract, NaCl	Production increase by 2.78-fold	[154]
*E. coli* BL21 (DE3)	Chitinase	Central Composite Design	Temperature, incubation time	Total activity increased by 1.54-fold	[115]
*E. coli* BL21(DE3)	Zinc metalloprotease	Central Composite Design	IPTG, Ca^2+^, induction time	Production increase by 15-fold	[22]
*E. coli* JM109	Carboxymethyl-Cellulose	Central Composite Design	Rice bran tryptone and initial pH of medium	Production increase by 3-fold	[155]
*P. pastoris* X33	Phytase	Central Composite Design	Yeast extract, tween-80, methanol	Specific activity increase by 21.8-fold	[156]
*E. coli* TB1	MBP-Heparinase	Central Composite Design (Orthogonal)	Yeast extract, glucose, Ca^2+^, OD600	Specific activity increase by 2.5-fold	[157]
*E. coli* BL21	*Cis*-epoxysuccinate hydrolase	Central Composite Design (Rotatable)	Inoculation level, induction-starting time, lactose, induction temperature, induction time	Enzyme activity increase by 4.6-fold	[158]

**Table 8:** Central Composite Design of four independent factors (labelled X_1_, X_2_, X_3_, X_4_ respectively) studied at two levels (+1 and −1) including two central point replicates (0 and 0). The table also shows different types of common responses found in optimisation process; (1) Actual data refers to experimental results; (2) predicted data are generated by software based on the design and actual results. The residuals are the difference between actual and predicted data.

Coded Values					Responses		
Runs	X_1_	X_2_	X_3_	X_4_	Actual	Predicted	Residuals
1	−1	1	−1	1	**Experimental response**	**Predicted response data**	**Residual data**
2	−1	−1	1	1			
3	0	0	0	0			
4	−1	0	0	0			
5	−1	1	1	−1			
6	1	1	1	1			
7	1	1	−1	1			
8	−1	1	1	1			
9	1	−1	−1	1			
10	0	−1	0	0			
11	1	1	1	−1			
12	0	0	0	0			
13	0	0	1	0			
14	0	1	0	0			
15	1	0	0	0			
16	0	0	0	1			
17	1	1	−1	−1			
18	−1	1	−1	−1			
19	−1	−1	1	−1			
20	−1	−1	−1	1			
21	1	−1	−1	−1			
22	0	0	0	−1			
23	1	−1	1	1			
24	0	0	−1	0			
25	1	−1	1	−1			
26	−1	−1	−1	−1			
					Responses (e.g., actual, predicted and residues) data are utilised during the optimisation analysis to evaluate the validity of the model and determine the optimum.		

**Table 9:** An example of Analysis of Variance (ANOVA) for Response Surface Methodology fitted to a second-order polynomial equation. The table depicts R-squared (*R*^2^), Adjusted R-squared (Adj-*R*^2^), Predicted R-squared (Pred-*R*^2^), degree of freedom (DF), adjusted sum of square (Adj SS), adjusted mean square (Adj MS), *F*-value and *p*-value of the model.

Source	DF	Adj SS	Adj MS	*F*-Value	*p*-Value
Model	11	40.4149	3.67408	1255.77	0.0001
Linear	4	3.1531	0.78828	269.43	0.0001
Square	4	35.3209	8.83022	3018.09	0.0001
Interaction	3	1.9409	0.64697	221.13	0.0001
Residues	40	0.117	0.00293		
Lack-of-fit	13	0.00369	0.00284	0.96	0.515
Pure error	27	0.0802	0.00297		
Total	51	40.532			
*R*^2^= 99.71%, Adj-*R*^2^ = 99.63%, Pred-*R*^2^ = 99.48%					

